# Mental Health Burden, Professional Shortage, and a Proposal to Utilize Task-Shifting Approach to Address Mental Health Needs in Remote Areas of Sindh Province, Pakistan

**DOI:** 10.1192/bjo.2025.10151

**Published:** 2025-06-20

**Authors:** Qammar Jabbar, Jawed Akbar Dars, Iqra Ansari, Manisha Kumari, Shaheryar Ali

**Affiliations:** 1 North Staffordshire Combined Healthcare NHS Trust, Stoke-on-Trent, United Kingdom; 2 National Institute of Child Health, Karachi, Pakistan; 3 Children’s Hospital Karachi, Karachi, Pakistan; 4 Civil Hospital, Mithi, Pakistan; 5 Jinnah Postgraduate Medical Centre, Karachi, Pakistan

## Abstract

**Aims:** This study aims to report the outcomes of mental health (MH) camps organized at various locations on a voluntary basis in District Tharparkar, an underserved area in the Sindh province of Pakistan. The camps aimed to screen, assess, educate, and treat patients with MH disorders. The study also reported the distribution of prevalent MH conditions in the Tharparkar area, barriers to attaining MH, and a possible solution to the MH professional shortage in remote areas.

Background: Mental disorders have become increasingly prevalent in Pakistan, affecting millions of people while there is a significant shortage of MH practitioners. MH camps play a crucial role in increasing awareness among the general population, reducing the stigma associated with MH conditions and offering an opportunity for the underserved population to approach MH professionals. Nonetheless, a more permanent solution is needed to address the needs of the patients in underserved remote communities.

**Methods:** A pre-structured Case Report Form was prepared to document the baseline characteristics, including family income, distance from a psychiatrist, duration of illness, perception regarding patients’ illnesses, healthcare visits in the last 6 months, and diagnosis of the patients.

**Results:** In 2022, a total of 460 patients were screened, and 164 (35.6%) were diagnosed with or considered for MH disorder. Out of these 164 patients, the mean age was 29.44 years (1–76). There were 94 (57.3%) males and 70 (42.7%) females. The average household income was PKR 25,758 (<£100), and the mean distance to a psychiatrist was 99 minutes, whereas the mean duration of presenting illness was 4.36 months. Depression was the most common illness reported (32%), followed by anxiety disorders (12%), psychotic disorders (9%), substance use (3%), and bipolar disorder (2%). Other mental health disorders were found in 28% of patients, and in 14%, diagnosis was not established.

**Conclusion:** More than half of patients sought help from a general practitioner in the last 6 months with the presenting complaints. The distance from the psychiatrist who serves as the primary care provider for MH needs in Pakistan and financial constraints are additional barriers for individuals residing in Tharparkar to attain help for MH needs. Therefore, task-shifting to general physicians (GPs) and incorporating MH into the primary care system could solve MH professionals’ deficiencies in remote areas. In light of the findings, it was recommended to initiate capacity-building sessions for the GPs in Sindh to train and educate them regarding the MH needs of their target population.

